# Behind Taxonomic Variability: The Functional Redundancy in the Tick Microbiome

**DOI:** 10.3390/microorganisms8111829

**Published:** 2020-11-20

**Authors:** Agustín Estrada-Peña, Alejandro Cabezas-Cruz, Dasiel Obregón

**Affiliations:** 1 Faculty of Veterinary Medicine, University of Zaragoza, 50013 Zaragoza, Spain; 2 UMR BIPAR, INRAE, ANSES, Ecole Nationale Vétérinaire d’Alfort, Université Paris-Est, 94700 Maisons-Alfort, France; alejandro.cabezas@vet-alfort.fr; 3 Center for Nuclear Energy in Agriculture, University of Sao Paulo, Piracicaba, São Paulo 13400-970, Brazil; 4 School of Environmental Sciences, University of Guelph, Guelph, ON N1G 2W1, Canada

**Keywords:** tick gut microbiome, functional redundancy, co-occurring bacteria

## Abstract

The taxonomic composition and diversity of tick midgut microbiota have been extensively studied in different species of the genera *Rhipicephalus*, *Ixodes*, *Amblyomma*, *Haemaphysalis*, *Hyalomma*, *Dermacentor*, *Argas* and *Ornithodoros*, while the functional significance of bacterial diversity has been proportionally less explored. In this study, we used previously published 16S amplicon sequence data sets from three *Ixodes scapularis* cohorts, two of uninfected nymphs, and one of larvae experimentally infected with *Borrelia burgdorferi*, to test the functional redundancy of the tick microbiome. We predicted the metabolic profiling of each sample using the state-of-the-art metagenomics tool PICRUSt2. The results showed that the microbiomes of all *I. scapularis* samples share only 80 taxa (24.6%, total 324), while out of the 342 metabolic pathways predicted, 82.7%, were shared by all the ticks. *Borrelia*-infected larvae lack 15.4% of pathways found in the microbiome of uninfected nymphs. Taxa contribution analysis showed that the functional microbiome of uninfected ticks was highly redundant, with, in some cases, up to 198 bacterial taxa contributing to a single pathway. However, *Borrelia*-infected larvae had a smaller redundancy with 6.7% of pathways provided by more than 100 genera, while 15.7–19.2% of pathways were provided by more than 100 genera in the two cohorts of uninfected ticks. In addition, we compared the functional profiles of three microbial communities from each data set, identified through a network-based approach, and we observed functional similarity between them. Based on the functional redundancy and functional similarity of the microbiome of ticks in different developmental stages and infection status, we concluded that the tick gut microbiota is a self-regulating community of very diverse bacteria contributing to a defined set of metabolic pathways and functions with yet unexplored relevance for tick fitness and/or bacterial community stability. We propose a change of focus in which the tick microbiome must be analyzed in all dimensions, highlighting their functional traits, instead of the conventional taxonomic profiling.

## 1. Introduction

The information on the microbial communities associated with parasitic arthropods is increasing dramatically mainly due to the availability of fast and affordable sequencing technologies [1,2,3,4]. The majority of the information concerns the taxonomic composition of the microbiome, while the functional significance of bacterial diversity has been proportionally less explored [5]. Recent studies bridged the information gap between taxonomy and function in microbiome research for a wide array of organisms [6,7]. Several studies have addressed the taxonomic composition of midgut microbiota in different tick species of the genera *Rhipicephalus*, *Ixodes*, *Amblyomma*, *Haemaphysalis*, *Hyalomma*, *Dermacentor*, *Argas* and *Ornithodoros* [1,2,3,4,5,6,7,8,9]. A highly diverse set of microbes has been identified in tick microbiota [10,11]. Notably, there is no consensus about the range (in terms of taxonomic variability) of the tick microbiome, and some studies report high [12] or poor [13] diversity. This is not surprising considering the many factors influencing tick microbiota composition which include, but are not limited to, physiological stress by environmental traits, blood meal, host species and developmental stage [14].

There is also evidence that the relation between tick-borne pathogens and tick microbiome is bidirectional. Experimental infection by some pathogens, such as *Anaplasma phagocytophilum*, was found to modulate microbiome diversity and composition [15] while perturbing the midgut microbiota with antibiotics reduced *Borrelia burgdorferi* colonization in ticks [16]. Some of these results have been validated in field studies. For example, field collected *Ixodes scapularis* with abundant midgut bacteria lack *B. burgdorferi* [8]. Despite the taxonomic variability observed in tick microbiome, the strong phylosymbiotic signal between *Ixodes* ticks and their microbial communities [3] suggests that tick-microbiota assemblies are not stochastic. Rather, the phylogenetic structure of microbial communities associated with ixodid ticks supports the existence of a species-specific tick holobiont with a largely unexplored influence on tick fitness and vector competence [3].

Phylosymbiosis does not necessarily presume that members of the microbial community are constant, stable or vertically transmitted; but rather to an eco-evolutionary pattern between the tick and the microbiome [17,18]. A hologenome (i.e., the collective genomes of the holobiont) can carry out many functions that result from the continuum of interactions of gene products between the tick and microbiota [19]. If the composition of tick microbiota assemblies is based on the selection of bacterial genomes that contribute to tick fitness, we can then hypothesize that the observed bacterial taxonomic variability features a functional background in which a predictable set of functions would be systematically recovered regardless of the taxonomic composition of the microbiomes. In a previous study, we found that communities of bacteria in tick microbiome are functionally redundant, suggesting that ticks select the microbiome that fulfills a core set of functions [20]. In addition, biological disturbance by *A. phagocytophilum* infection, or the Tick Antifreeze Glycoprotein (IAFGP), altered the relative proportion of several bacteria modifying the structure of the tick microbiome. Pathogen infection and antimicrobial peptide had however minor and no change, respectively, on the functional profile of the tick microbiome [20]. In this study, we assessed the metabolic pathways profile of the microbiome of three cohorts of *I. scapularis*, one of engorged larvae experimentally infected with *B. burgdorferi* and two of uninfected nymphs, aiming to demonstrate that the screening of the tick microbiome in terms of its taxonomic composition and variability is simplistic, and underestimates the multidimensionality of the hologenome, especially its functional traits. We demonstrate that despite differences in developmental stage and infection status, different communities of bacteria contain coherent profiles of metabolic pathways. Therefore, inferences based on the taxonomic profile are often lacking biological significance. Instead, we propose an integrative approach for the analysis of the tick microbiome, reinforcing their metabolic capabilities, the microbial interaction patterns and the complementarity of taxonomical and functional analyses. Such improvement is expected to contribute to our further understanding of tick microbiota, in terms of microbiome variation between tick species, seasonal variability of the tick microbiome, interactions among bacterial taxa or the changes induced by physiological stress of ticks.

## 2. Methods

**Figure 1:**
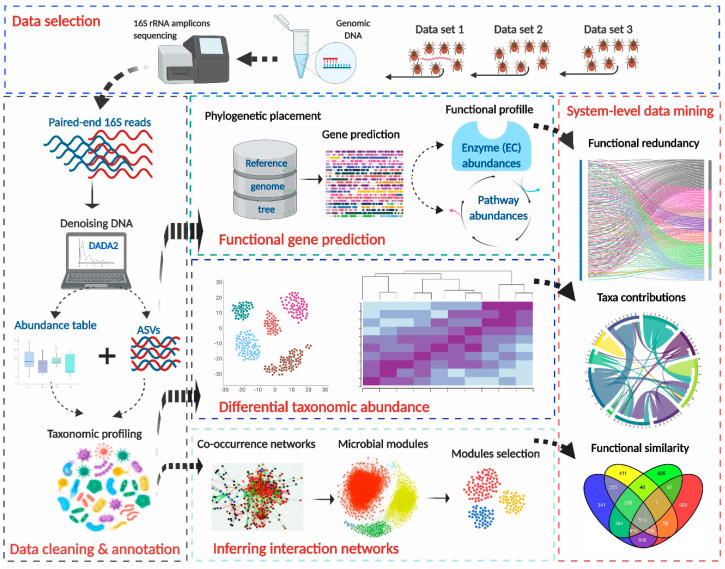
Workflow illustrating the analytical steps of the study. Three data sets were selected from published studies on the microbiome of *Ixodes scapularis*, fed on mice, from similar lab conditions and protocols. Raw data were obtained from available repositories online, consequently, were processed following the QIIME2 pipeline, i.e., were denoised and identified at the levels of amplicons sequence variant (ASV) and annotated taxonomically based on SILVA 132 database. The ASV profiles and sequences were used to predict the metabolic profile from those microbiomes, based on the bioinformatic tool PICRUSt2. In addition, the taxonomic profiles were compared using the statistical method ALDEx2. The taxonomic profiles were also used to infer co-occurrence networks, from which three modules were extracted in each data set and used to predict their functional profiles. Finally, integrative analyses were performed, linking taxonomic and functional profiles, to assess functional redundancy and functional similarity in each data set.

We used published 16S rRNA gene (16S) sequencing data on the microbiomes of engorged larvae and nymphs of *Ixodes scapularis* ticks. These data sets were generated by the same research group using comparable methodologies and were obtained by amplicon sequencing bacterial 16S rRNA gene, using barcoded universal primers (515F/806R) and sequenced on an Illumina MiSeq system that generated 250-base paired-end reads. The raw data were obtained in the frame of three different experiments: (1) the study by Narasimhan et al. [21], including engorged tick larvae that were infected under laboratory conditions with *Borrelia burgdorferi* s.s. and pooled in groups of 5 before DNA extraction. For our study, we used the ’control’ data set generated from larvae that fed on ovalbumin-immunized and *B. burgdorferi*-infected mice (*n* = 24), (2) the report by Abraham et al. [22] studying the gut microbiota composition of individual nymphs after infection with *Anaplasma phagocytophilum* (we used the control data set from uninfected ticks, *n* = 10) and (3) one data set produced by Abraham et al. [22] about the effects of an antimicrobial peptide on the gut microbiota (we used the control group of nymphs treated with a mock peptide, *n* = 12). In short, this study roots on three different experiments (hereinafter referred to as “Data set 1”, “Data set 2” and “Data set 3”) carried out on the same tick species, which were kept under the same environmental (laboratory) conditions and sequenced with the same protocols and technology. The complete workflow described below is schematically outlined in Figure 1.

### 2.1. Processing of Original Raw Sequences

We performed *de novo* taxonomic annotation of all the 16S data sets. To this end, the sequences were downloaded from SRA repository [23], extracted and de-interlaced in two fastq data sets containing only the first or second mate read [24] using the data analysis platform Galaxy (http://usegalaxy.org). Demultiplexed fastq files were pre-processed and analyzed using QIIME2 pipeline (v. 2019.1) [25]. Briefly, the DADA2 software package [26] was used (via q2-dada2) for denoising the fastq files, merging mate reads and chimera removal using the consensus method, which is the equivalent to “default parameters”. The amplicon sequence variants (ASVs) were annotated using the q2-feature-classifier plugin classify-sklearn naiïve Bayes taxonomy classifier [27] against the 16S SILVA database (release 132) [28].

### 2.2. Prediction of Functional Traits in Tick Microbiome

The 16S amplicon sequences were used to predict the metabolic profiling of each sample. PICRUSt2 [29], a robust bioinformatic tool, was used to predict the genomes from 16S amplicon sequences. The ASVs were placed into a reference tree (NSTI cut-off value of 2) containing 20,000 full 16S sequences from prokaryotic genomes, which is then used to predict individual gene family copy numbers for each ASV. The predictions are based on several gene family catalogs, like Kyoto Encyclopedia of Genes and Genomes (KEGG) orthologs (KO) [30], Enzyme Classification numbers (EC) [31]. Pathway profiles were inferred from structured pathway mapping based on MetaCyc database [32]. Different from other databases, like SEED subsystem [33] and COG [34], the MetaCyc database does not organize the functional features into standard hierarchical categories (i.e., four or five levels of resolution). Instead, some pathways can be collapsed into four organization levels in MetaCyc, whereas other pathways can be classified at ten levels. To deal with this, we explored the entire set of pathways in MetaCyc, and collapsed them into four main levels of organization of functions and metabolic processes, hereafter referred to as “pathways > L3 > L2 > L1” (Supplementary Material S1) and that hierarchical classification was applied to the functional profile of each data set in this study.

### 2.3. Differential Taxonomic Composition Analysis

For assessment of differential abundance of taxa, we adhered to published methodology [34] for relative abundance analysis of proportional data using ALDEx2 algorithm. It estimates variation within each sample using Monte-Carlo instances drawn from the Dirichlet distribution, which maintains the proportional nature of the data and returns a multivariate probability distribution. The method uses the centered log ratio (clr) transformation; this ensures that data are scale invariant and coherent among data sets. The ALDEx2 tool performs parametric and non-parametric (Kruskal—Wallis) statistical tests on the clr values, and reports the differences with effect-size estimates, and *p* values adjusted for multiple testing with Benjamini–Hochberg correction [35]. The analysis was performed with the package ALDEx2 [36] on the R programming environment [37]. We obtained more than 600 ASVs after the de novo taxonomic annotation. The data sets were filtered (i.e., rare ASVs with less than 10 total reads and detected in less than 30% of samples of each data set were removed) and then collapsed at genus level, obtaining 324 features on which the ALDEx2 pipeline was applied. Venn diagrams (using the online tool at http://bioinformatics.psb.ugent.be/webtools/Venn/) were used to calculate the number of genera shared by the three data sets (hereafter ubiquity analysis).

We also performed a Detrended Canonical Analysis (DCA) using the taxonomic profile of each data set, looking for the ordination of the individual tick specimens of the three data sets into coherent groups (i.e., each sample is assigned to the right data set based on their taxonomic composition). The hypothesis is that a large separation of the three data sets in the ordination space would be indicative of differential composition on bacterial communities (presence/absence/abundance) in data sets expected to be highly similar. Similarly, we prepared Venn diagrams and carried out a DCA with the functional pathways predicted for each data set, with the same hypothesis. The R package vegan [38] was used for multivariate analyses.

### 2.4. Testing Functional Redundancy of Tick Microbiome

Functional redundancy is a property of most microbial systems, which implies that each metabolic function can be performed by a wide range of coexisting and taxonomically distinct organisms [39]. In this study, we explore the unrevealed functional redundancy of the tick functional microbiome. The purpose was (i) to check the functional redundancy of the pathways, identifying the pathways according to high or low redundant functions in the tick microbiome, (ii) to evaluate if the same taxa persistently contribute to each pathway in the different data sets. The relative importance of each pathway was evaluated according to the number of individual contributing genera, defined here as “Degree of redundancy”. Linkages between taxonomic profiling and functional profiles were achieved from the PICRUSt2 metagenome predictions, using the function “Taxa contribution”. For data analysis purposes, the ASV profile was collapsed at genus level, while the pathways profiles were analyzed at the levels of both pathways and L3 functional processes (Supplementary Material S1).

### 2.5. Co-Occurring Bacteria and Functional Similarity

Functional similarity is the ability of two microbial communities to carry out a functional process at a similar rate, regardless of differences in composition [40]. We hypothesize that functional redundancy in tick microbiome underlay the relationships between microbial taxa, and the filtering of taxa in living microbial communities, as an evolutive specialization of the tick holobiont to prevail at changing environmental conditions. To test this hypothesis, we computed the modules (i.e., communities) of co-occurring bacterial genera in each data set. The aim was to demonstrate that probabilistic modules of co-occurring taxa from the same data set represent biological arrangements of selected microbes that provide a consistent set of metabolic pathways. These sets would persist under changing combinations of bacterial taxa. For each data set, we calculated the SparCC-correlations [41] between the number of reads of pairs of co-occurring organisms, removing those between the range −0.3 to +0.3 because these are considered to be non-significant correlations. We built networks of co-occurring taxa and calculated their modularity by the Louvaine algorithm [42] using Gephi 0.92 (available at www.gephi.org, accessed October, 2019). In our application, modularity represents probabilistic modules of taxa that tend to co-occur more frequently among them than with other taxa. We selected for further procedures the three larger modules of taxa emanating from the network of each data set. We translated the modules of co-occurring taxa in each data set to their functional profile and also explored the taxa contribution to the predicted metabolic pathways.

## 3. Results

### 3.1. B. burgdorferi Infection and Developmental Stage Are Associated with Changes in Taxonomic Composition and Bacterial Abundance

**Figure 2:**
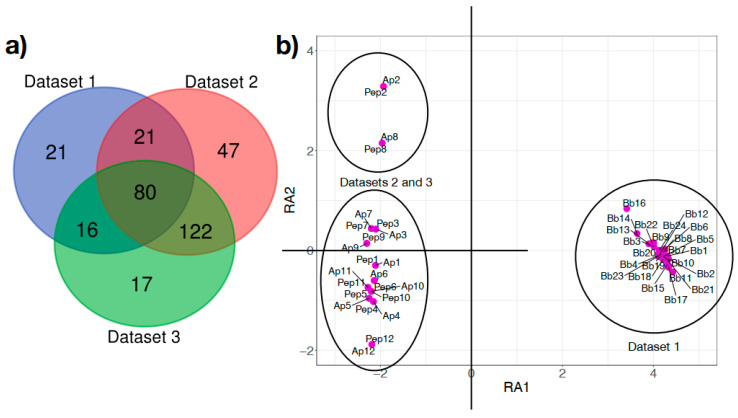
The microbiome of *Ixodes scapularis* ticks shares few taxa among ticks. (**a**) Venn diagram of the number of shared bacterial genera in the three sample groups of *Ixodes scapularis.* Data sets 1 to 3 consist, respectively, of engorged tick larvae infected with *Borrelia burgdorferi* s.l. (Data set 1), nymphs serving as control (i.e., uninfected) in a study of infection with *Anaplasma phagocytophilum* (Data set 2) and nymphs inoculated with a mock peptide used as a control in an experiment with an anti-microbial peptide (Data set 3). (**b**) Plot of the samples of each of the three data sets, separated by a Detrended Canonical Analysis along the bacterial genera detected in each tick. Groups are labeled with the name of the data set and each tick sample (each dot) labeled with letters and a continuous numbering (Bb: Data set 1; Ap: Data set 2; Pep: Data set 3). RA1 and RA2 are the axes in the reduced space after the canonical analysis, explaining the distance among samples and data sets.

The ubiquity analysis revealed that only 80 bacterial genera (out of 324, 24.6%) were shared by all the three data sets (Figure 2a). Only 117 genera (36.1%) were shared by *Borrelia*-infected engorged larvae and uninfected nymphs. A total of 122 genera (37.6%) were shared by both uninfected sets of nymphs. A total of 21 (6.5%), 47 (14.5%) and 17 (5.2%) genera were identified in only one of the data sets (Figure 2a). To further evaluate the differences between the microbiota of infected and uninfected ticks, we carried out a multivariate ordination of the taxonomic profile found in each sample. The DCA shows that the differences in bacterial taxonomic composition were informative enough to clearly separate infected larvae from uninfected nymphs (Figure 2b), but DCA on taxonomic grounds lacks resolutive power to separate the two cohorts of uninfected nymphs.

**Figure 3:**
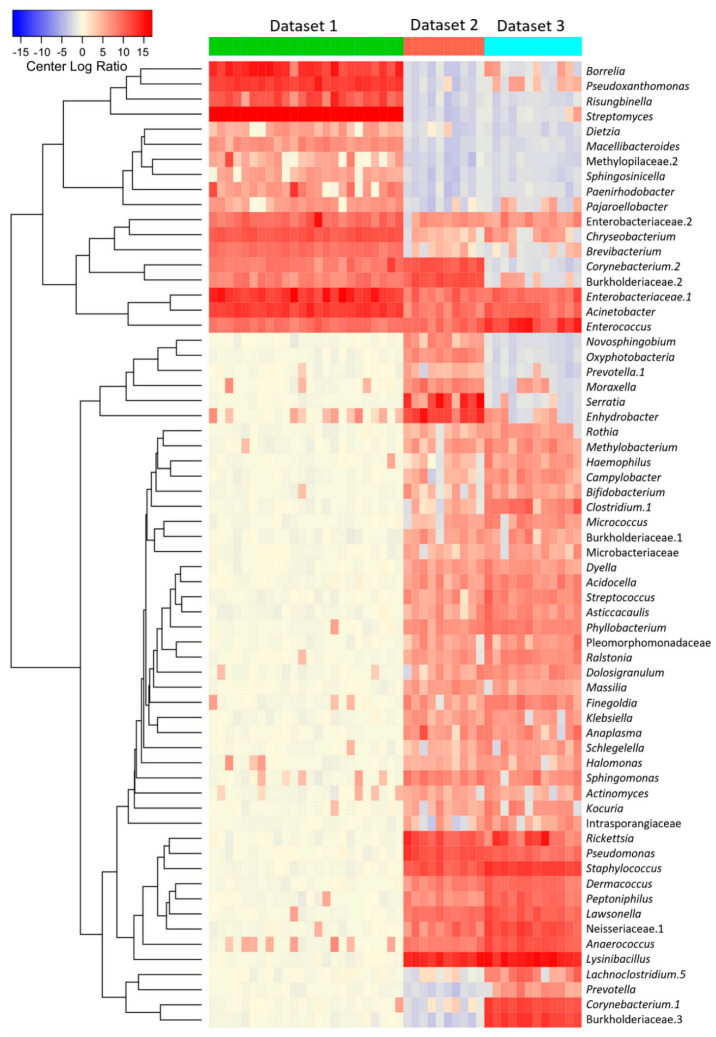
The microbiome of *Ixodes scapularis* ticks shows bacterial taxa with highly significant differences in relative abundance. The figure shows a heatmap derived from the center log ratio (clr) abundance values of the 60 top bacterial genera in terms of significant differences among the three data sets, as detected by the ALDEx2 algorithm, using a Benjamini—Hochberg correction of *p*-values (Reiner (Kruskal—Wallis, *p* < 0.001).

We then tested whether the abundance of the bacterial genera was different between groups. Figure 3 shows the relative abundance (clr transformed values) of the 60 top bacterial taxa with the highest significant differences among the three data sets, as detected by the ALDEx algorithm (Kruskal—Wallis, *p* < 0.001). The abundance of bacteria was clearly different between infected engorged larvae and uninfected nymphs. Minor differences in abundance were observed among uninfected ticks of the Data sets 2 and 3. The complete taxonomic profiling of bacteria genera with significant differences in abundance is included in Supplementary Material S2.

### 3.2. Lower Functional Diversity in the Microbiome of Borrelia Infected Larvae Compared to Uninfected Nymphs

**Figure 4:**
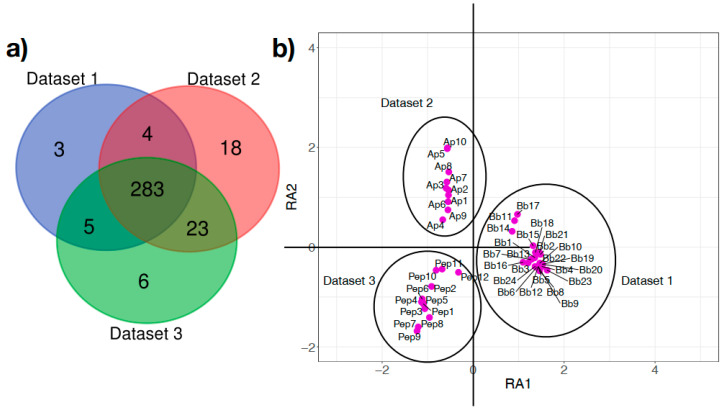
The microbiome of *Ixodes scapularis* ticks shares a high proportion of functional pathways among tick specimens: (**a**) Venn diagram of the number of shared functional pathways in the three data sets of *Ixodes scapularis*. Data sets are the same and labeled in a similar way to those in Figure 2. (**b**) Plot of the samples of each of the three data sets, separated by a Detrended Canonical Analysis along the taxonomic profile detected in each tick sample. Groups are labeled with the name of the data set and each tick sample (each dot) labeled with letters and a continuous numbering (Bb: Data set 1; Ap: Data set 2; Pep: Data set 3). RA1 and RA2 are the axes in the reduced space after the canonical analysis, explaining the distance among specimens and data sets.

The functional metabolic profiling resulting from PICRUSt2 genome prediction produced a total of 342 metabolic pathways in the three data sets (Figure 4a). Of these, a core of 283 (82.7%) pathways was recorded in the microbiome of every tick in the three data sets). A total of 330 pathways (96.5%) were shared by uninfected nymphs, whereas tick microbiomes of *Borrelia*-infected larvae differed from the others in 21 and 9 metabolic pathways, respectively (6.1 and 2.6%). *Borrelia*-infected larvae lack 15.4% of pathways present in uninfected nymphs. However, it is out of the scope of this manuscript to elucidate whether missing pathways are associated with *Borrelia* infection or developmental stage. A DCA carried out using the metabolic pathways of the three data sets produced three close, but well-differentiated, groups of ticks in accordance to their respective data sets (Figure 4b). These results suggest that the functional profiling of the microbiome of *I. scapularis* provide better information for comparative purposes than the taxonomic profiles since functional traits are highly conserved. The only 3 pathways that appeared only in infected engorged larvae were detected at most in one or two ticks; we therefore consider that this is not a significant result.

### 3.3. Taxa Diversity Contributes to Similar Functional Profiles in Tick Microbiome Regardless of Borrelia Infection and Developmental Stage

**Figure 5:**
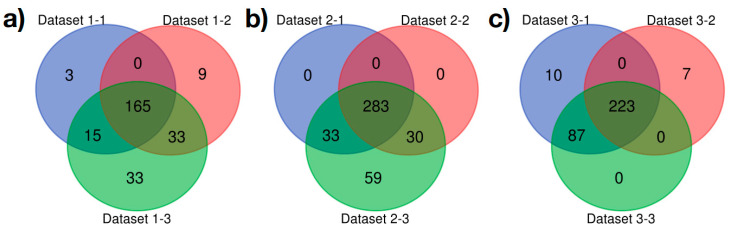
Clusters of co-occurring bacterial taxa in *Ixodes scapularis* have similar functional profiles. The Venn diagrams show the number of shared metabolic pathways among three modules (communities) of bacterial genera in each data set. The modules were derived from the networks of co-occurring bacteria in each data set. Each group of three modules per data set contains together more than 90% of the total bacterial genera identified in each data set. (**a**) Data set 1; (**b**) Data set 2; (**c**) Data set 3.

We then tested the contribution of taxa diversity within microbial communities to the functional traits of the tick microbiome. To this aim, we inferred networks of co-occurrence, computed the probabilistic modules of most common co-occurring taxa that can be regarded as microbial communities and predicted the pathways profile for the top 3 modules (including at least 90% of bacterial taxa) in each data set. Networks of *Borrelia*-infected larvae produced 7 modules, and uninfected nymphs in Data sets 2 and 3 formed 3 and 5 modules, respectively. The comparison of the number of metabolic pathways shared among network modules of each data set is summarized in Figure 5. The level of functional similarity among modules of each data set is high. The 3 larger modules of bacteria in infected engorged larvae represented 258 functions (total 63%, total 405). A similar result was found in uninfected nymphs of Data set 3 as only 327 pathways (80%, total 405) were predicted to occur in the 90% of taxa conforming to these 3 modules. The three modules of bacterial genera in Data set 2 together encompassed the complete set of 405 pathways, being functionally identical. Therefore, networks resulting from co-occurring taxa in each data set are different in terms of bacterial taxa (nodes in a module do not occur in other), but they result in similar contributions to the functional profile.

### 3.4. Functional Pathways Are Highly Redundant in the I. scapularis Microbiome

**Figure 6:**
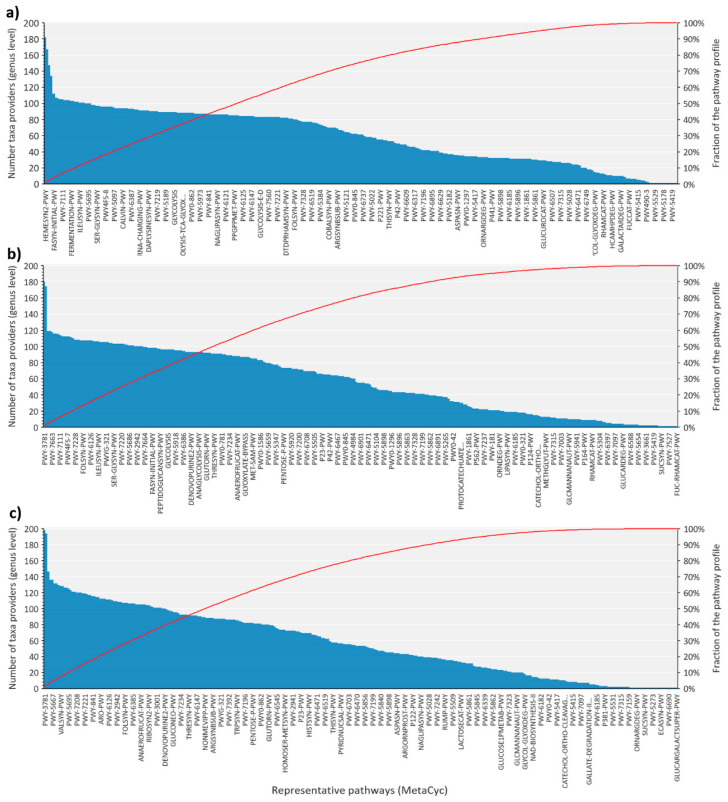
The number of bacterial taxa (collapsed at genus level) that contribute to the predicted metabolic pathways in the microbiome of *Ixodes scapularis*. The charts include the number of genera as histograms (left *y*-axis) that contribute to each metabolic pathway (*x*-axis), together with the fraction of the predicted pathway profile (continuous line, right *y*-axis). (**a**) Data set 1; (**b**) Data set 2; (**c**) Data set 3.

The results above suggested that several bacteria within tick microbiota could carry out similar functions. To further test this hypothesis and to evaluate the functional redundancy of tick microbiome, we performed an analysis of taxa contribution to metabolic pathways (Figure 6). The analyses revealed that approximately 90% of metabolic pathways are carried out by 20 or more bacterial genera. Notably, up to 167, 174 or 198 bacterial genera may contribute to the most redundant pathways in *Borrelia*-infected larvae and uninfected nymphs of Data sets 2 and 3, respectively. Up to 20 (6.7%), 52 (15.7%) or 61 (19.2%) functions are predicted to be represented by more than 100 bacterial genera in each data set, respectively. This result suggests that the functional diversity of the microbiome in *Borrelia*-infected larvae is slightly reduced in terms of functional redundancy compared to the microbiome of uninfected nymphs.

**Figure 7:**
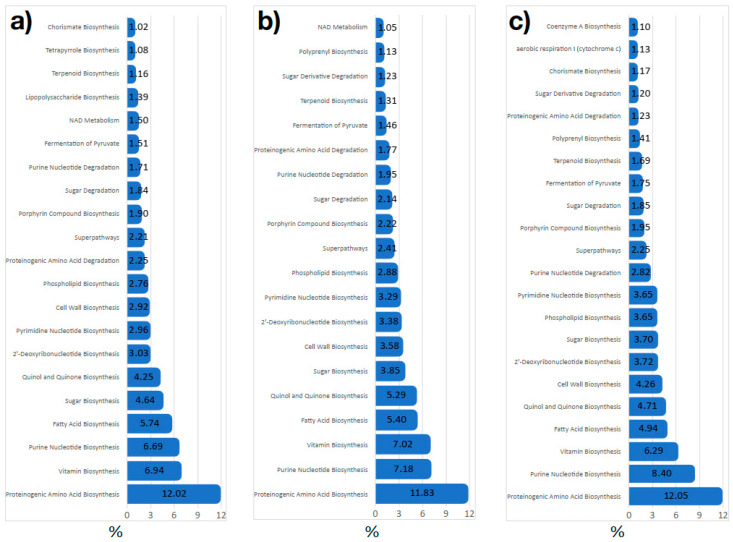
The percent of functional processes represented in the microbiome of *Ixodes scapularis*. The bars show the functional processes (from MetaCyc pathways maps, collapsed at L3, according to Supplementary Material S1) represented in each of the three data sets of microbiomes of *I. scapularis*. Only L3 processes represented as more than 1% of the total of each data set are displayed. (**a**) Data set 1; (**b**) Data set 2; (**c**) Data set 3.

The large number of metabolic functions makes it difficult to capture the persistence of the functional microbiome of *I. scapularis*. For further exploration of functional traits, the pathways were collapsed as functional processes of the L3 category (according to Supplementary Material S1) to overview the processes most represented in each data set. Figure 7 displays the L3 processes with more than 1% of representation in each data set. The three data sets contained the same number of L3 processes with similar values of representativity for each process. These results suggest that several bacterial genera contribute to different pathways involved in L3 processes. As a proof-of-concept, we show the taxa contribution to metabolic pathways involved in the L3 process “Cofactor and Vitamin biosynthesis” (Supplementary Material S3). Different bacterial genera have been identified in each data set (a, b, c for Data sets 1, 2 and 3, respectively), contributing differently to the metabolic pathways, but resulting in a coherent set of functional L3 level processes. Both pathways and processes profiles are similar among the three data sets.

## 4. Discussion

Herein we demonstrated that a functional core exists in the tick gut microbiome regardless of developmental stage and infectious status. This functional core results from the assemblages of co-occurring groups of bacteria that, independently of their taxonomic identity, contribute to a defined set of functions. We wanted to test whether such a functional profile is persistent among individual ticks, do not randomly change in the same species and stage of tick, and whether its variability is a good marker to differentiate tick cohorts. Our approach can also capture the effects of the infection by pathogens, since it is known that some pathogens alter the tick microbiome [8,22,23]. We used as a proof-of-concept the previously published and publicly accessible microbiomes of three groups of ticks of the same species (*I. scapularis*) kept under the same laboratory conditions and processed using the same sequencing methodology. Identification of bacterial taxa is commonly based on 16S amplicon sequences. However, bacterial abundance analysis using this method is limited due to the maximum number of reads to record, and the variable number of copies of the 16S region to be amplified. To overcome these issues, we used the centered log ratio (clr) of the number of reads [35] and computed co-occurrence patterns among taxa by the SparCC correlation [42]. Both methods ensure that data are scale invariant and are comparable among data sets.

Two important conclusions emanated from our results. Firstly, as previously reported, we found high taxonomic variability in the microbiome of the different cohorts of ticks. For example, only 24.6% of taxa were identified simultaneously in the three data sets. After the removal of the *Borrelia*-infected ticks, that introduced the highest bias, only 37.6% of taxa were shared. The low resolution of the taxonomic microbiome to discriminate cohorts of uninfected ticks suggests that taxa comparisons are not optimal in studies comparing the gut microbiome of uninfected ticks. In contrast to the uninfected cohorts, the DCA using the taxonomic microbiome discriminated uninfected and infected ticks, which suggests that taxonomic comparisons may be informative in the context of pathogen infection. The predicted functional microbiomes showed small variability at pathway level among data sets. Secondly, our results support the stability of the functional microbiome despite known changes in taxonomic composition due to *Borrelia* infection [21,22] and developmental stage [14]. Comparison of the data sets revealed differences in *Borrelia* infected larvae in terms of (i) a lower occurrence and diversity of bacteria, (ii) a lower functional redundancy and (iii) a lack of coherence in the network built around co-occurring taxa. However, the 47 missing metabolic pathways in *Borrelia*-infected larvae represent less than 1% of the total number of reads in the data set. It is beyond the focus of this study to individually analyze each missing pathway, but we can foresee a field of research in the resolution of the importance of each pathway for the tick’s physiology.

We provided evidence of functional redundancy in the tick microbiome, a pattern previously found in the microbiome of several metazoan [43,44,45,46], including ticks [20]. Some pathways resulted as highly redundant, with up to 198 bacterial genera contributing to a single pathway. We interpret this as proof of the importance of highly redundant pathways for either the microbiome or for the tick fitness, since it is now well established that some bacteria are necessary for tick physiological resilience [47,48]. The functional microbiome is composed by different taxa that co-occur at different proportions in the microbiome at the individual tick level. Taxonomic variability has been reported in several studies of the tick microbiome, but here we showed that within the diverse array of bacterial taxa, the metabolic capabilities of the tick microbiome persist. Once grouped at functional processes level L3, all the microbiomes resulted in similar functional patterns in terms of presence/absence and proportion in each data set. This is the main argument for which, in our view, the tick gut microbiome must be considered an entity in comparative studies, extending the taxonomic compositional analysis with an approach that disentangles the microbial interaction patterns, and fundamentally their functional traits.

The functional redundancy observed in all the data sets in this study was also supported by the data of the microbial communities inferred by the modules of co-occurring taxa. This is a new finding in the research of tick microbiome: the functional microbiome of the tick gut results from the co-occurrence of bacteria at apparently random combinations but producing a coherent functional profile, independently of its taxonomic composition. Networks describe interactions among co-occurring entities and have been applied to a variety of fields [41]. Networks have been already used to inquire about the relationships of ticks collected under different ecological conditions and their microbiome [20]. The importance of network constructs is that they emanate an important property, the so-called modularity. It is a probabilistic context that displays modules of bacteria co-occurring more frequently among them than with others. Modules are mutually exclusive since a taxon cannot belong to two modules; therefore, they culminate in the “most probable” arrangements of taxa.

This is consistent with the concept that habitat filtering could be the major driving force for structuring microbiomes [3,5,18,30]. Functional similarity in tick’s gut microbiome appears from discrete functional groups of bacterial taxa that co-occur with similar and redundant metabolic capacity. This phenomenon has been reported in the microbiomes of arthropods and implies strong environmental filtering in shaping their structure [40,45,46,49,50]. Our results support that the tick gut microbiome is a self-regulating community of bacteria, aimed to keep the homeostasis of its metabolic profile, other than the bacteria already recognized as true endosymbionts of ticks [49]. How this could correlate with findings of bacteria in field-collected ticks [51,52] is yet unaddressed. It did not escape our attention that the functional concept outlined here also opens a door to a rational design of vaccines against the tick’s microbiome, aiming for vaccines targeting metabolic features, like functional proteins and pathways with low redundancy or that are of central importance for tick’s physiology.

This study was primarily aimed to develop a proof-of-concept supporting the unreliability of the classical approaches of comparing compositional constructs for the analysis of the tick microbiome. Instead, we demonstrated that the analysis of functional traits (e.g., predicted pathways in this study), in combination with the analysis of microbial interaction patterns, constitute a highly coherent approach for these purposes. The conclusion is that the tick taxonomic microbiome is an assemblage of bacteria that results in a homeostatic combination of functional features. Our interpretation of these results is that (i) microbiomes of ticks from the same species do not share a high proportion of bacterial taxa, (ii) metabolic pathways profiles are highly conserved, although slightly reduced in *Borrelia*-infected larvae, (iii) different modules of co-occurring bacterial taxa contribute in similar ways to the complete set of pathways (i.e., warrant functional similarity), (iv), metabolic functional processes profiles (L3) are identical in every data set.

Most probably, it arises by yet unknown mechanisms of selection of co-occurring bacterial taxa, that complements a core set of functional pathways required for the self-maintenance of the microbiota and presumably for the tick. We propose this framework as a standard tool for future comparative studies among tick microbiomes avoiding the simplistic view based on lists of bacteria.

## Supplementary Materials

The following are available online at https://www.mdpi.com/2076-2607/8/11/1829/s1. Supplementary Material S1: Tabulation and collapsing of the metabolic pathways features according to the MetaCyC database (metacyc.org), classified at four hierarchical categories of functional processes (pathways > L3 > L2 > L1), as compiled by one of the co-authors (DO). Supplementary Material S2: The significant differences among the centered log ratio (clr) values of each bacterial genera found in the microbiomes of *Ixodes scapularis* using the ALDEx2 algorithm. Each tick sample belonging to a single data set is included with labeling “Bb” (Data set 1), “Ap” (Data set 2) and “Pep” (Data set 3) and consecutive numbering. Bacterial taxa with a blue background show significant differences between the three data sets studied. Sheet “Selected” contains the 104 bacterial genera selected for the preparation of the heatmap in Figure 3. Supplementary Material S3: Alluvial charts displaying a proof-of-concept regarding the contribution of a very variable taxonomic microbiome to the core functional microbiome of *Ixodes scapularis*. Charts shown in (**a**), (**b**) and (**c**) correspond respectively to the Data sets 1, 2 and 3, and include the bacterial genera involved in the metabolic route of “Cofactor and Vitamin biosynthesis”. Each taxon (left bars) contributes differently to each metabolic pathway (bars in the center of each chart) and then to a similar set of L3 processes (right). The width of each band corresponds to the sum of the contribution of each taxon to each specific pathway and process. Functions and taxa contributions were inferred using PICRUSt2 full pipeline [29].
