# Supplementary figures and images for: Behind Taxonomic Variability: The Functional Redundancy in the Tick Microbiome
Source: Microorganisms. 2020 Nov 20;8(11):1829. doi: 10.3390/microorganisms8111829 (PMC7699746; doi:10.3390/microorganisms8111829)

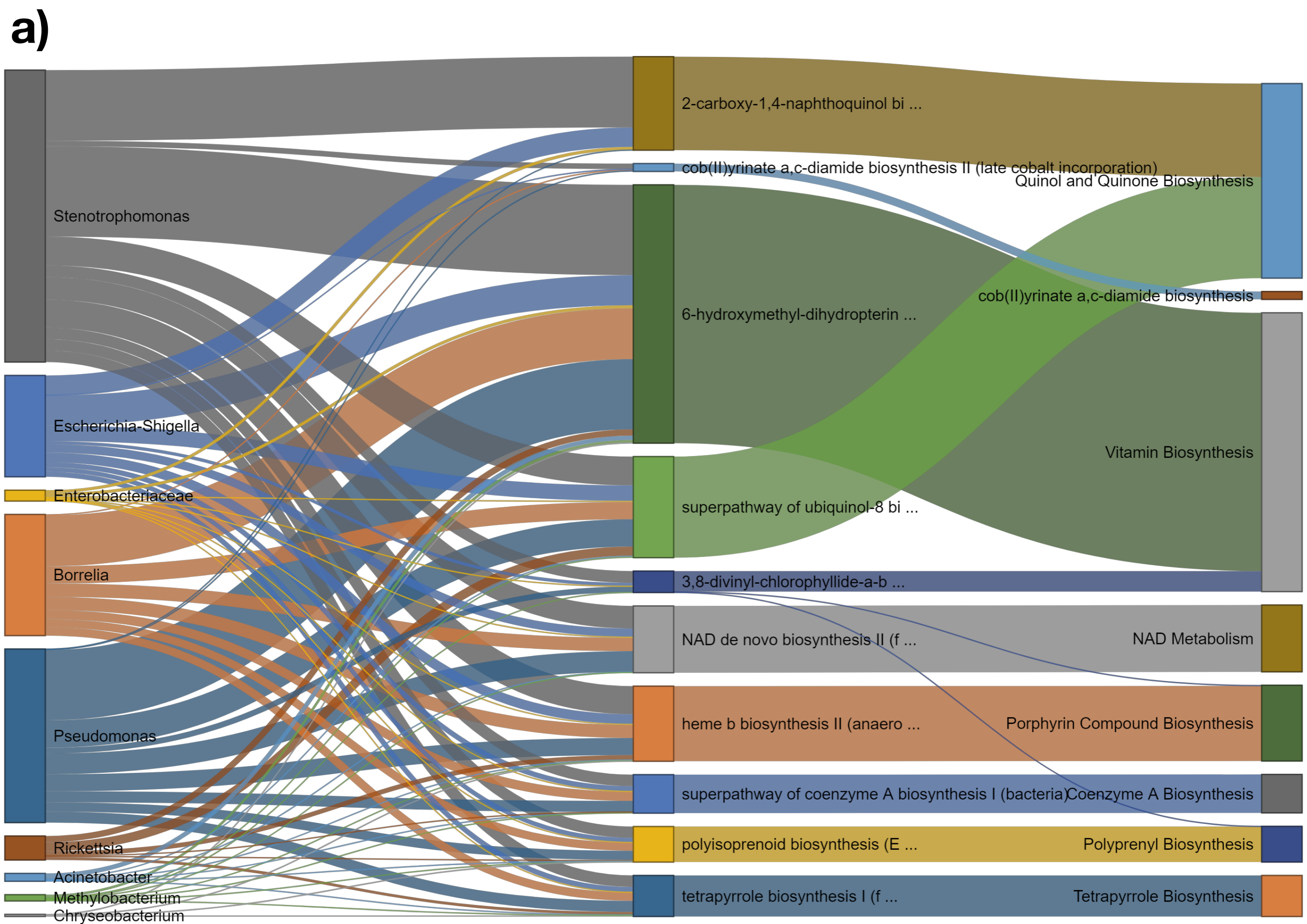

**A**

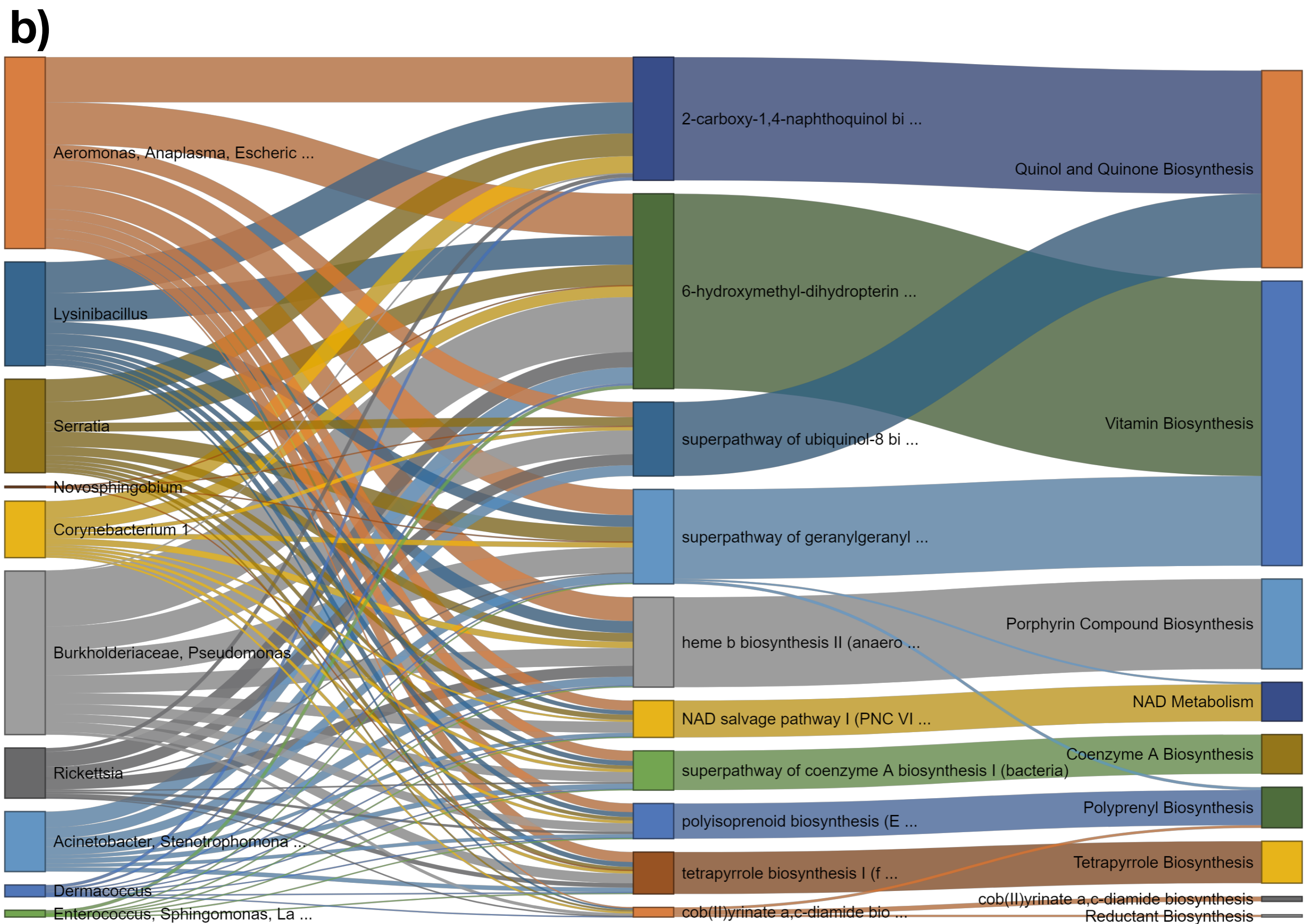

**B**

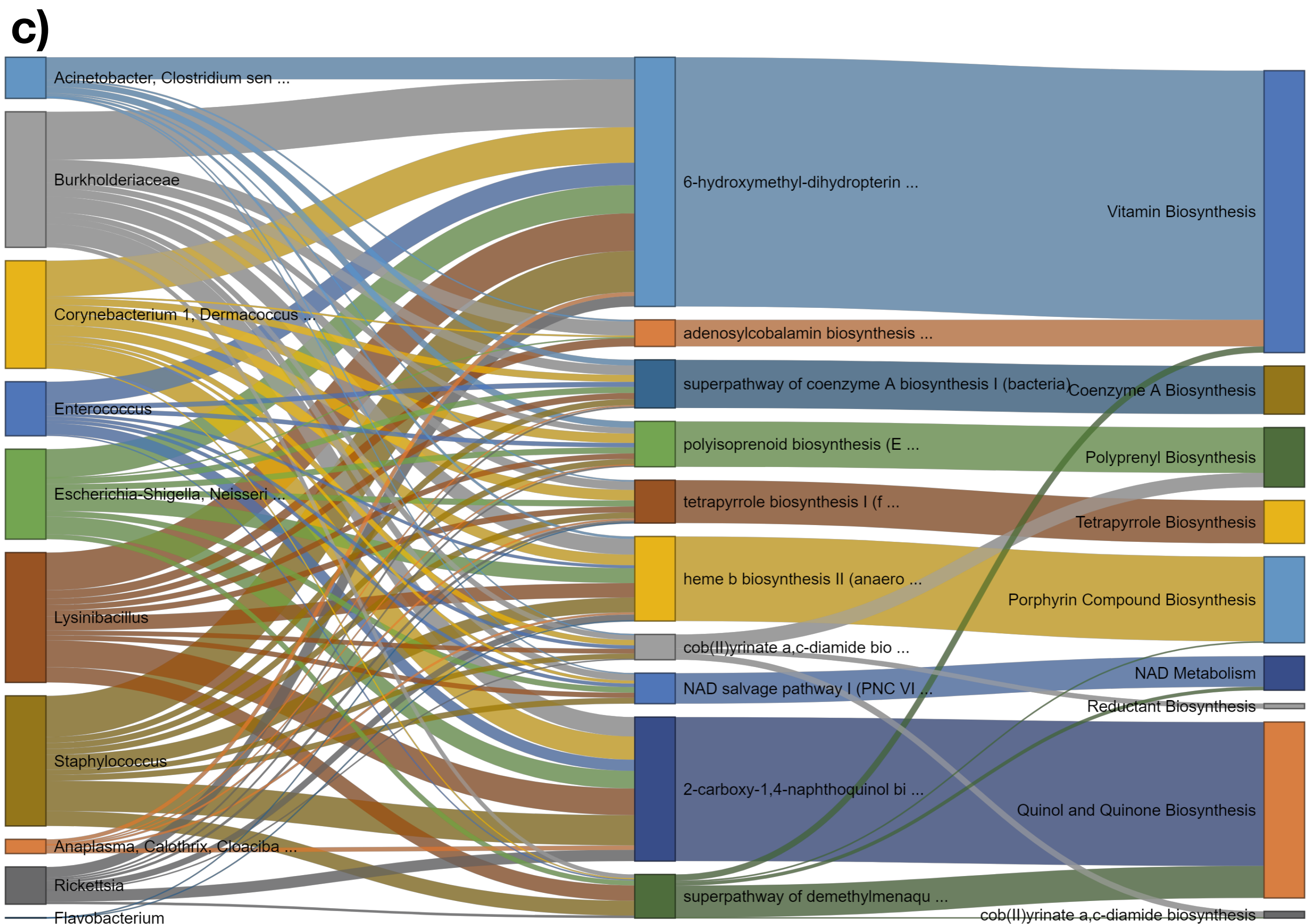

Supplement: Supplementary file 1 [file microorganisms-08-01829-s001.zip › Supplementary Material 3.pdf]
